# High Performance Liquid Chromatography–Tandem Mass Spectrometry Method for Correlating the Metabolic Changes of Lactate, Pyruvate and L-Glutamine with Induced Tamoxifen Resistant MCF-7 Cell Line Potential Molecular Changes

**DOI:** 10.3390/molecules26164824

**Published:** 2021-08-10

**Authors:** Ala A. Alhusban, Sokiyna Albustanji, Lama A. Hamadneh, Aliaa I. Shallan

**Affiliations:** 1 Department of Pharmacy, Faculty of Pharmacy, Al-Zaytoonah University of Jordan, Amman 11733, Jordan; sukainabustanji94@gmail.com (S.A.); lama.hamadneh@zuj.edu.jo (L.A.H.); 2 Department of Analytical Chemistry, Faculty of Pharmacy, Helwan University, Cairo 11435, Egypt; alia.shallan@pharm.helwan.edu.eg

**Keywords:** HPLC–MS/MS, metabolic changes, breast cancer, Tamoxifen resistance, MCF-7

## Abstract

Breast cancer is one of the most prevalent cancers worldwide usually treated with Tamoxifen. Tamoxifen resistance development is the most challenging issue in an initially responsive breast tumor, and mechanisms of resistance are still under investigation. The objective of this study is to develop and validate a selective, sensitive, and simultaneous high performance liquid chromatography–tandem mass spectrometry method to explore the changes in substrates and metabolites in supernatant media of developed Tamoxifen resistance MCF-7 cells. We focus on the determination of lactate, pyruvate, and L-glutamine which enables the tracking of changes in metabolic pathways as a result of the resistance process. Chromatographic separation was achieved within 3.5 min. using a HILIC column (4.6 × 100 mm, 3.5 µm particle size) and mobile phase of 0.05 M acetic acid–ammonium acetate buffer solution pH 3.0: Acetonitrile (40:60 *v*/*v*). The linear range was 0.11–2.25, 0.012–0.227, and 0.02–0.20 mM for lactate, pyruvate, and L-glutamine, respectively. Within- and between-run accuracy was in the range 98.94-105.50% with precision (CV, %) of ≤0.86%. The results revealed a significant increase in both lactate and pyruvate production after acquiring the resistant. An increase in L-glutamine levels was also observed and could be attributed to its over production or decline in its consumption. Therefore, further tracking of genes responsible of lactate, pyruvate, and glutamine metabolic pathways should be performed in parallel to provide in-depth explanation of resistance mechanism.

## 1. Introduction

Breast cancer (BC) is the most commonly diagnosed cancer globally with an estimation of 11.7% incidence of all cancer cases [1]. The incidence and mortality among diagnosed cases in females worldwide were 24.2% and 15%, respectively [2]. Tamoxifen (TMX), the most well-known selective estrogen receptor modulator, has been utilized frequently as an adjuvant therapy for hormone receptor (HR)-positive BC [3]. It shows high efficacy in reducing the risk of BC recurrence and related mortality [4]. Despite the importance of using TMX in the treatment of BC, most patients develop chemoresistance with time and relapse to more aggressive BC [5,6]. Hence, different mechanisms have been suggested to explain the resistance including the loss of estrogen receptor (ER) expression [7], pharmacological tolerance and alteration in cellular kinase/transduction pathways [8], modification and alteration of some regulatory proteins [9], and increased expression of epidermal growth factor receptor (EGFR) and human epidermal growth factor receptor-2 (HER2) [10]. However, other mechanisms are still unknown [11]. Therefore, different in vitro models have been investigated to further understand the resistance mechanisms. From which, MCF-7 cell line, an ER-positive progesterone receptor (PR)-positive cell line, represents the most commonly used cell line in BC research for TMX resistance [12].

Lactate dehydrogenase (*LDH*) is involved in the conversion of pyruvate to lactate and vice versa, concomitantly with the inter-conversion of NAD^+^ and NADH [13]. Experiments in our laboratory by Hamadneh et al. [14], showed a significant increase in *LDH-B* gene expression during the development of TMX resistance in MCF-7 cells up to 3-fold with gradual increase of TMX doses which is consistent with other published research [15]. Increased *LDH-B* gene expression led to a significant increase in lactate level and expected to show a further increase in pyruvate level. Likewise, Glutamate-ammonia ligase (*GLUL*) exhibited a significant gene overexpression up to 25-fold during TMX-resistance development with gradually increased doses of TMX which may lead to increased endogenous L-glutamine synthesis [16]. Thus, simultaneous monitoring of lactate, pyruvate, and L-glutamine level changes can be very helpful in understanding TMX resistance, and provide deeper insights to the pathology, biomarker discovery, and drug development in BC treatment.

Metabolomic approaches aim to understand cellular behavior by covering the metabolic profile for better explanation of any metabolic changes or reprogramming that occurs in cells as a response to any external or internal stimuli such as diseases, environmental changes, or treatment by drugs [17]. Several techniques have been applied to quantify metabolites such as enzymatic assays and analytical methods. However, the use of enzymatic assays is time-consuming, usually specific for one compound, and are highly affected by natural factors like temperature [18] and lack accuracy and precision [19]. Both Mass Spectrometry (MS) [20] and NMR [21] provide more accurate quantifications of different metabolites. However, complex matrices require the utilization of sophisticated separation techniques. For example, capillary electrophoresis has been used for metabolites monitoring in cell culture [22,23,24,25] and for profiling of amino acids in human urine samples [26], gas chromatography coupled with MS has been employed to identify feasible metabolic biomarkers permitting early diagnosis of lymphomas [27]. However, HPLC-MS [28], and HPLC tandem MS [29,30] have shown higher sensitivity and more powerful analysis capabilities.

The objective of this study was to develop, validate, and apply a new bio-analytical method for simultaneous determination of lactate, pyruvate, and L-glutamine in supernatant media of acquired TMX resistance MCF-7 cells using gradually increased doses of TMX and correlate the normalized metabolites levels with cells number with possible molecular changes. This will enable the conducting studies on a larger scale.

## 2. Experimental

### 2.1. Chemicals and Materials

TMX powder was purchased from (Santa Cruz Biotechnology, Dallas, TX, USA), and dissolved in dimethyl sulfoxide (DMSO) from (ChemCruz™ Biochemicals, Dallas, TX, USA), to prepare the required doses for treating and developing resistance in MCF-7 cells. Acetic acid, ammonium acetate, and acetonitrile were purchased from (Sigma Aldrich, St. Louis, MO, USA) and were used for the preparation of the mobile phase. Lactic acid, pyruvic acid, and L-glutamine were obtained from (Sigma Aldrich, St. Louis, MO, USA) and were used for the preparation of standard solutions.

### 2.2. MCF-7 Cells Culturing Process

MCF-7 (HTB-22™) cell line is a type of BC cell lines obtained from a 69-year-old Caucasian woman with pleural effusion metastatic BC. It is the most widely BC cell line used as a xenograft model and characterized by expression of both ER and PR. MCF-7 cell line with passage number (9) was obtained from American Type Culture Collection (ATCC) and cultured in triplicates following the standard protocol described here: the culture media consisted of RPMI medium (EuroClone, S.p.A., Via Figino, Italy) without L-glutamine with addition of 10% Fetal Bovine Serum (FBS) (EuroClone, S.p.A., Via Figino, Italy), 1% L-glutamine, 1% penicillin-streptomycin and 1% 4-(2-hydroxyethyl)-1-piperazineethanesulfonic acid (HEPES) with total volume of 20 mL. MCF-7 cells were cultured in T-75 flask and incubated with 95% humidity, 5% CO_2_ at 37 °C in an incubator (Memmert Incubator Oven INB200, Germany). Cells’ confluence and morphology were checked by ZOE Fluorescent Cell Imager (Bio-Rad, Hercules, CA, USA).

#### 2.2.1. TMX Treatment

TMX powder was dissolved in DMSO to prepare the required doses. The percentage of DMSO used to dissolve TMX was less than 0.0008% to avoid any toxic effect from DMSO to the cells. Once the cultured cells in the flasks reached 70% confluency, they were treated over the culturing time with different doses of the prepared TMX. Exposure to TMX was increased gradually starting from 0.1 µM up to 40 µM. Cells were first treated with 0.1 µM TMX for 3 days, then the media containing TMX was removed and replaced with fresh media to allow the cells to grow to 70% confluency. The next TMX treatment would follow for 3 more days with gradual increase in TMX concentration until the cells were treated with 40 µM. MCF-7 cells toxicity from TMX were evaluated previously and showed very low apoptotic response after treatment with TMX [16].

#### 2.2.2. Media Collection, Cells Density, and Counting Process

The counting process of the cultured MCF-7 cells were performed before and during TMX treatments, the prepared media were collected during each step of cell counting as follows: The supernatant media (20 mL) was carefully removed from the T-75 cell culture flasks, filtered through a 0.22 µm filter, collected in falcon tubes, and stored at nominal temperature of −70 °C until LC-MS/MS analysis. The cultured MCF-7 cells were washed in the flask with 3 mL of phosphate-buffered saline (EuroClone, S.p.A., Via Figino, Italy) to remove any dead cells without affecting the pH of the residual live attached cells, and then discharged into a waste bottle. For these live cells, Accutase (EuroClone, S.p.A., Via Figino, Italy) was used for the detachment process by the addition of 1.5 mL of the Accutase solution to the flasks containing the cells and were incubated for 5 min. at 37 °C. After incubation, the flasks were gently shaken to ensure total detachment of the cells from the flasks’ surface, followed by adding 3.5 mL of the prepared RPMI-1640 media to the T-75 flasks over the cells and Accutase solution, to terminate any harmful action of Accutase on cells. The whole solution consisted of MCF-7 cells, Accutase solutionz and the added media, and was wholly collected in falcon tubes and centrifuged using 1100 RPM at 4 °C for 7 min. to separate the cells from the residual solutions. As a result of centrifugation, the cells formed a pellet at the bottom of the tube, and the formed pellets were re-suspended by addition of 5 mL of the prepared RPMI-1640 followed by gentle shaking up and down to demerge the clustered cells. Using 1 mL of the suspended pellets for cell counting by Hemocytometer and Compound binocular light microscope (Nikon, Tokyo, Japan), the other 4 mL of suspended pellets were split, and 1 million cells were seeded in new T-75 flasks with freshly prepared RPMI-1640 medium. After the cells’ attachment, they were treated with the next TMX dose.

### 2.3. HPLC-MS/MS Method Development

Preparation of calibration standards and samples for chromatographic analysis: Standard stock solutions were prepared by dissolving each analyte in Milli-Q water, and then diluted to prepare working standard solutions. Serial dilution was performed to prepare a series of at least five concentration levels each repeated three times for each analyte. The concentration levels were 0.1124, 0.2247, 0.5618, 1.6854, and 2.2472 mM for lactate, 0.0119, 0.0244, 0.056, 0.113, and 0.227 mM for pyruvate, and 20, 60, 100, 120, 160, and 200 µM for L-glutamine in addition to blank samples.

Samples for analysis were prepared by taking 100 µL of each of the collected supernatant media and diluted at (1:9) ratio with Milli-Q water, mixed well and filtered through a 0.22 µm syringe filter, then injected into HPLC-MS/MS system for the analysis. No guard column was used.

#### 2.3.1. Instrumentation and Conditions

Chromatographic analysis was performed using (ExionLC, AB SCIEX, Foster City, CA, USA) HPLC system equipped with ABDXR5370003, ABDXR5370002 pump, ABCXR5370001 autosampler, AB2CT5370010 Column oven, ABCBM5370044 Controller and ABDG5370039 Degasser. Mass spectrometric detection was carried out using a triple quadrupole mass spectrometer with atmospheric pressure ionization (API) 4500 (ABSciex, Ontario, Canada) operated in positive or negative electrospray ionization and multiple reaction monitoring (MRM) mode. Hardware control, data acquisition and treatment were carried out using Analyst 1.63 Software (ABSciex, Concord, Canada). The ion transitions were monitored at *m*/*z* 89.000 → 43.100, *m*/*z* 87.000 → 43.000 and *m*/*z* 147.000 → 84.000 for lactate, pyruvate, and L-glutamine, respectively.

**Figure 1:**
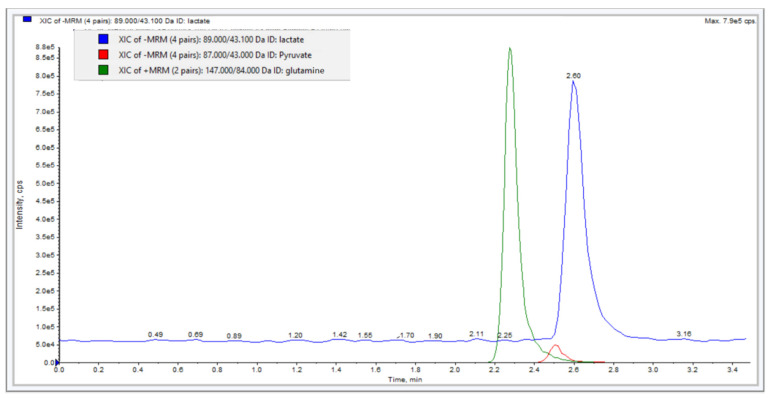
A chromatogram showing the chromatographic separation under optimized conditions; HILIC column (4.6 × 100 mm, 3.5 µm particle size) and mobile phase of 0.05 M Acetic acid–ammonium acetate buffer solution pH 3.0: Acetonitrile (40:60 *v*/*v*). Flow rate at 0.6 mL/min, and Injection volume of 5 μL. The analytes of interest are shown; Blue: 3.5 mM Lactate (off-set), Red: 0.38 mM Pyruvate, and Green: 0.2 mM L-Glutamine.

Due to the hydrophilic and polar nature of these ions, we employed a hydrophilic interaction liquid chromatography (HILIC) column for separation. Chromatographic separation was achieved within 3.5 min. using XBridge^®^ HILIC column (4.6 × 100 mm, 3.5 µm particle size) and mobile phase of 0.05 M Acetic acid–ammonium acetate buffer solution pH 3.0: Acetonitrile (40:60 *v*/*v*). Isocratic elution at flow rate of 0.6 mL/min. was used and the temperature of the column oven was 40 °C. The injection volume was 5 µL. The detector optimized conditions were as follow: the nebulizer gas was air with zero grade, and nitrogen was used as the auxiliary. The source/gas-dependent parameters were 20 psi curtain gas, 8 psi collision gas, 500 °C medium temperature, 4500 V ion spray voltage, and 40 psi ion source gas one and two. Negative ionization mode was used for the detection of lactate and pyruvate and positive ionization mode was used for L-glutamine. The chromatogram under optimized conditions is presented in Figure 1.

#### 2.3.2. HPLC-MS/MS Method Validation

The analytical method was validated according to the EMA guideline for bioanalytical method validation [31]. The obtained results from the validation procedure including linearity, recovery, matrix effect, dynamic range, limit of detection (LOD), and quantification (LOQ) in addition to selectivity, accuracy and precision, and stability have been used for the judgment on the reliability, consistency, and quality of the applicable analytical method.

External calibration standards of at least 6 points were used to construct the calibration curves and establish the linear range. The prepared standard solutions were injected into the system. The retention time was recorded and the area under the curve (AUC) were automatically calculated. Selectivity and specificity were verified by injecting purified water and three samples of RPMI-1640 culture media; to detect any peaks present within the range of retention time of the analytes of interest. Within- and between-run accuracies were calculated and evaluated at six replicates of four QC levels (0.15, 0.35, 1.00, and 2.50 mM lactate), (0.015, 0.035, 0.10, and 0.25 mM pyruvate), and (30, 80, 180, and 280 µM L-glutamine) for all analytes of interest in one analytical run and three runs in three different days, respectively. Within- and between-runs precision were estimated by running six samples to ensure the repeatability and reproducibility of the used method for all analytes. Both LOD and LOQ were calculated based on the standard deviation (SD) of intercepts of the calibration curves (σ) and the slope of the calibration curves (S) for each analyte standards (n = 3). The stability of samples at room temperature was evaluated by preparing three samples of the culture media at two QC levels for each analyte and analyzing them after storing at room temperature for 8 h against a calibration curve. Freeze–thaw stability for one cycle was also evaluated by preparing sufficient amounts of the three samples at two QC levels for each analyte and analyzing them against a calibration curve.

## 3. Results and Discussion

### 3.1. MCF-7 Cells Morphological Change

**Figure 2:**
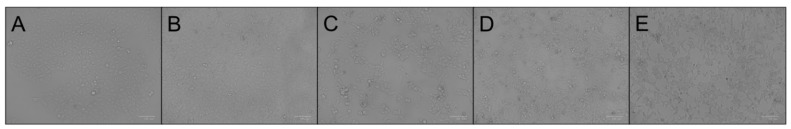
Changes in MCF-7 cells morphology during the treatment with increased gradual doses of TMX, (**A**): Control (no treatment), (**B**): treatment with 0.5 µM TMX, (**C**): treatment with 10 µM, (**D**): treatment with 35 µM, and (**E**): treatment with 40 µM.

MCF-7 cells are characterized as an epithelial-like shape having strong cell-to-cell adherent properties and well-organized as monolayer [32]. A noticeable change in MCF-7 cells morphology was observed during the TMX treatment operation as shown in Figure 2. The observed morphological changes were aligned with changes presented in our previous works [14,16]. The significant changes in cells morphology have been seen at 35 µM. The cells lost their monolayer epithelial shape to become more rounds and aggregates in 40 µM treatment. This morphological change includes the transition from the epithelial phenotype to mesenchymal phenotype which contribute to changes in cellular shape of TMX resistant MCF-7 cells. Moreover, Bui et al. have reported that as a result of acquiring resistant to TMX in MCF-7 cells, an overexpression of mesenchymal marker proteins was observed, contributing to cells morphological transition from epithelial phenotype to mesenchymal phenotype in a process known as epithelial–mesenchymal transition (EMT) [33]. EMT was also stimulated by other inducers such as melanoma cells adhesion molecule (MCAM), in which, significant expression of this inducer is highly related to poor survival of BC patients and specially in those who were treated with TMX only [34]. All of these factors play a role in the morphological change of the resistant MCF-7 cells that was noticeable in this work.

### 3.2. Cell Counting and Cell Density

**Figure 3:**
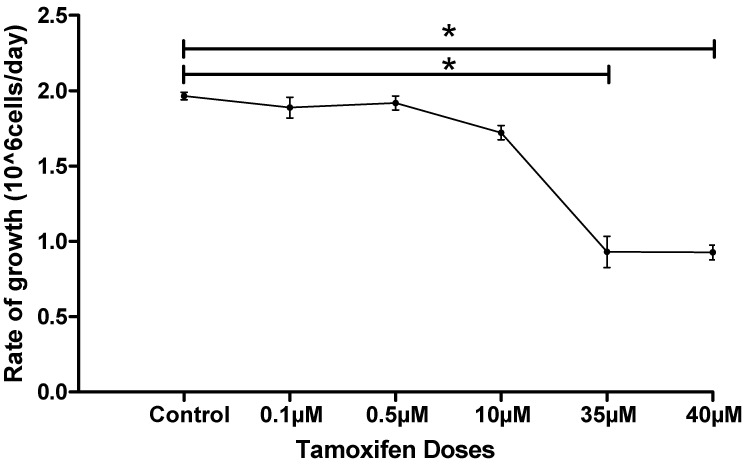
Rate of growth of MCF-7 cells over developing acquired TMX resistant cells throughout treatment with TMX in different doses increased gradually. Statistical significance was calculated by one way ANOVA followed by Tukey hoc test in GraphPad prism 5.0 software, considering the statistically significant as next: (* significant at *p* ≤ 0.05) results were expressed as mean ± SD (n = 3 runs for each sample).

The numbers of treated MCF-7 cells were counted and an increase in the rate of growth of the developed resistant TMX cells was observed after treatment with 35 µM. This was also reported by Knowlden et al. which presented an elevation in the proliferation of TMX resistant MCF-7 cells [35]. Table 1 shows MCF-7 cells density before and during the treatment with gradual increase of TMX doses. MCF-7 cells density was calculated by counting live MCF-7 cells in 1 mL of RPMI-1640 media (10^6^ cells/1 mL) by Hemocytometer for each culturing cycle, and the rate of growth was measured accurately following the same period of culturing. Data graphing was accomplished by GraphPad Prism 5.0 software as shown in Figure 3. The rate of growth of treated cells with TMX decreased around the concentration of 35 μM and 40 μM, which is the IC_50_ reported in our study in MCF-7 cells sensitive to TMX [14], and only cells that started to have molecular and metabolic changes survived the increased concentrations of TMX in comparison to control and low TMX dose groups.

### 3.3. HPLC-MS/MS Method Validation

All parameters including the separation column stationary phase, mobile phase composition, flow rate and pH value, injection time and volume, separation temperature, and detector conditions were carefully optimized for the analysis of lactate, pyruvate, and L-glutamine simultaneously in supernatant media of MCF-7 cell line. Injecting solvent and blank showed no interference peaks at the retention time for lactate, pyruvate, and L-glutamine. In addition, no carry-over or significant suppression or enhancement of signals detection were reported for any of the analytes. Moreover, short-term freeze–thaw stabilities for one cycle was evaluated and found to be stable with accuracy values at the range within 7% of the nominal concentration. Other validation parameters were calculated and summarized in Table 2. The method is accurate and robust as indicated by within- and between-runs accuracy and precision values. The recovery percentages were in the range of (99.85–100.34%) and the response was linear over the selected calibration ranges, R^2^ = 0.9972, 0.9963, and 0.9988, for lactate, pyruvate, and L-glutamine, respectively. Therefore, the need to introduce internal standard to samples, which is used mostly to minimize errors resulting from sample processing, was not required as the obtained validation results were successful and method required no extraction procedure and minimal sample pretreatment steps in addition to avoid the possibility of analytes loss resulting from addition of other substances.

### 3.4. Determination of Lactate, Pyruvate, and Glutamine in Cell Culture Supernatant

**Figure 4:**
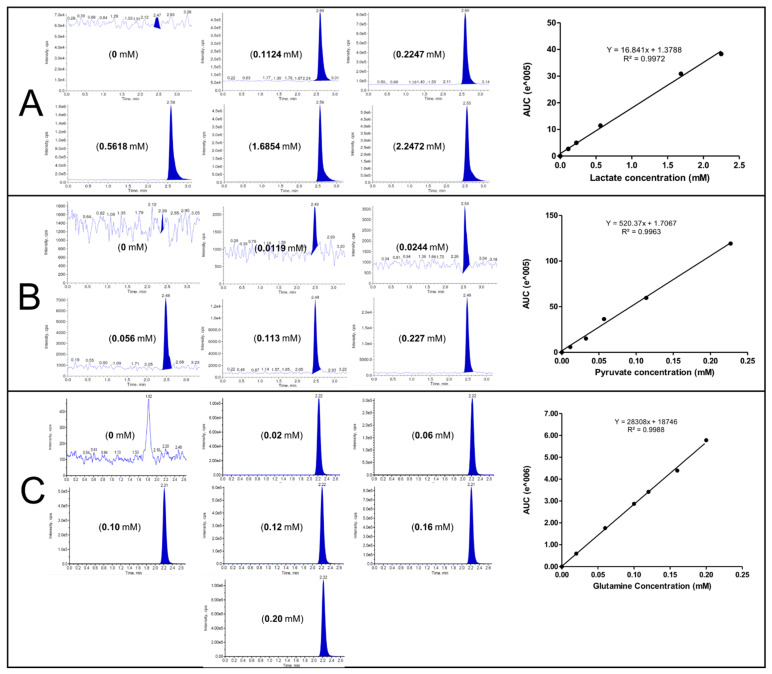
Chromatograms of analytes by HPLC-MS/MS used for calibration curves construction, using at least 6 concentration levels under optimized chromatographic conditions (mentioned in Figure 1). (**A**): Lactate at M/Z (89.000/43.100 Da) negative mode. (**B**): Pyruvate at M/Z (87.000/43.000 Da) negative mode. (**C**): L-glutamine at M/Z (147.000/84.000 Da) positive mode.

**Figure 5:**
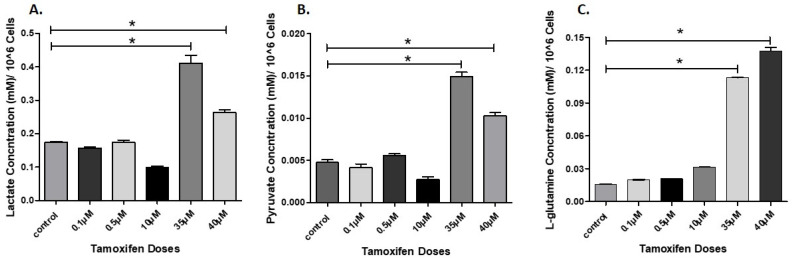
Calculated concentrations by HPLC-MS/MS of produced (**A**): lactate, (**B**): pyruvate and (**C**): L-glutamine from MCF-7 cells supernatants after treatment with TMX in different doses increased gradually. Statistical significance was calculated by one way ANOVA followed by Tukey hoc test in GraphPad prism 5.0 software, considering the statistically significant as next: (* significant at *p* ≤ 0.05) results were expressed as mean ± SD (n = 3 runs for each sample from 3 culture flasks).

The calibration curves for lactate, pyruvate, and L-glutamine by the developed HPLC-MS/MS method under optimized conditions were constructed using at least six concentration levels of standard solutions dissolved in Milli-Q water as shown in Figure 4. Samples were then analyzed and the analyte concentration was calculated using the equation from the corresponding calibration curve and corrected with cell count of 1 million cells as shown in Figure 5.

A significant increase in lactate production was observed after treatment with 35 µM of TMX Figure 5A. This can be due to either Warburg effect in resistant MCF-7 cells [36], and/or the increased *LDH-B* enzyme expression that has a key role in TMX resistant MCF-7 cells [14,37]. For pyruvate, a little fluctuation in its production was noticed before treatment with 35 µM TMX. While significant increase was observed after treatment with 35 µM and 40 µM of TMX Figure 5B. This can also be attributed by the overexpression of *LDH-B* that was reported in our laboratory by Hamadenh et al. after developing TMX resistant MCF-7 cells, and consequently a significant increase in concentration of produced pyruvate in addition to lactate [14]. Furthermore, EMT process that noticed in TMX resistant MCF-7 cells was accompanied by an increase in the expression of *LDH-B* isoenzyme that is responsible for the conversion of lactate to pyruvate and vice versa, even though the absence of expression in some cancer types of BC and prostate cancer due to hypermethylation of the promoter in genes level [38]. As mentioned before the significant increase of both lactate and pyruvate may occur as a result of up-regulation of different metabolic pathways due to developing resistance and cellular adaptation to hypoxia environment in BC [39].

In the case of L-glutamine, the cell culture supernatant contained detectable amount before treating the cells with TMX. After calculating the existed concentration of L-glutamine in the supernatant media, the concentration was corrected with cell count of one million MCF-7 cells as presented in Figure 5C. L-glutamine concentration levels increased upon treatment with TMX, which could be either due to a decline in consumption from the added L-glutamine to the prepared RPMI-1640 media, or an increase of production due to up-regulation of some genes responsible for L-glutamine synthesis by inducing specific enzymes such as glutamine synthetase [16]. In addition, a significant change in the rate of consumption was noticeable especially after treatment with 35 µM of TMX, which was around 8-fold compared to control and low TMX dose treatment groups. L-glutamine has important roles not only as an energy source for cells, but also in contributing to fighting acid stress that result from the Warburg effect and over production of lactic acid, and consequently helps in the growth of resistance MCF-7 cells [40]. As reported by other investigators, an overexpression of L-glutamine synthetase was observed in luminal phenotype but not in basal phenotype cells. The up-regulation of glutamine synthetase and down-regulation of glutaminase was induced by some genes in luminal phenotype BC cells [41]. MCF-7 cells were used in this work which are luminal phenotype cells. So, the noticeable and significant changes in L-glutamine concentration in supernatant media may be due to up-regulation in glutamine synthetase enzyme expression [16] or down-regulation of glutaminase enzyme expression, which could be a hallmark in developing resistant MCF-7 cells in the future.

## 4. Conclusions

A new bioanalytical assay was conducted by the developed and validated HPLC-MS/MS method. The analysis was made for supernatant media of developed acquired TMX resistant MCF-7 cells to track the changes in selected metabolic pathways as a result of the resistance process. There were significant changes in metabolites such as lactate, pyruvate, and L-glutamine. A significant increase in lactate and pyruvate production were detected after acquiring the resistant and EMT process happened after treatment with 35 µM of TMX and gaining the IC_50_. Meanwhile, L-glutamine concentration in supernatant media increased from the beginning of developing the resistance process, but a significant increase was observed after treatment with 35 µM of TMX. The increased concentration of L-glutamine may occur as a result of over production or a decline in consumption. Therefore, tracking all genes responsible for L-glutamine metabolic pathways can be an aim of the future study to provide further explanation.

Some light could be thrown on different aspects that may be helpful in future work, such as tracking changes in numerous biological compounds like glucose, other organic and amino acids, and DNA sequencing. This detection provide very valuable information helps in targeted metabolic profiling that could reveal the hidden aspects in many types of cancer and helps in well understanding of the resistance problems and cellular behaviors which are still challenging for many researchers.

## Figures and Tables

**Table 1:** Starting and ending MCF-7 cells density (10^6^ cells/1 mL RPMI-1640 media) and changes over treatment with increased gradual doses of Tamoxifen (µM) in the sequential interval of times (Mean ± SD, n = 3).

Tamoxifen Dose	Average of Started MCF-7 Cells Density (10^6^ Cells/5 mL)	Average of Ended MCF-7 Cells Density (10^6^ Cells/5 mL)
Control	1.000	2.965 ± 0.95
0.1 µM	1.000	2.888 ± 0.69
0.5 µM	1.000	2.919 ± 0.47
10 µM	1.000	2.721 ± 0.51
35 µM	1.000	1.930 ± 0.93
40 µM	1.000	1.936 ± 0.49

**Table 2:** Summarized results of chromatographic method validation parameters. With regard to limit of detection (LOD), limit of quantification (LOQ), linearity (R^2^), analytes concentration within specific linearity range (µM), within-run and between-run accuracy and precision (RSD %) for multi number of injections (n) and percent recovery.

Parameter	Lactate	Pyruvate	L-Glutamine
LOD (µM)	0.16	0.28	4.6
LOQ (µM)	0.49	0.849	13.95
R^2^	0.9972	0.9963	0.9988
Linear Range (mM)	0.11–2.25	0.012–0.227	0.02–0.20
Within-run Accuracy % (n = 6)	102.53	99.26	104.31
Between-run Accuracy % (n = 6)	104.11	98.94	105.50
Within-run precision (RSD%, n = 6)	0.39	0.32	0.48
Between-run precision (RSD%, n = 6)	0.61	0.77	0.86
Recovery %	99.85	100.34	100.33

## Data Availability

The data presented in this study are available on request from the corresponding author.

## Sample Availability

Samples of the compounds are commercially available.
